# Incorporation of the Altered Cast Technique in the Fabrication Workflow of an Implant-Assisted Removable Partial Denture (IARPD) for an Elderly Patient

**DOI:** 10.1155/2023/5249889

**Published:** 2023-08-16

**Authors:** Dimokritos Papalexopoulos, Theodora-Kalliopi Samartzi, Panagiotis Tsirogiannis, Ioli-Ioanna Artopoulou, Nikitas Sykaras

**Affiliations:** Department of Prosthodontics, School of Dentistry, National and Kapodistrian University of Athens, Zografou, Greece

## Abstract

Implants are currently used to serve as abutments for implant-assisted removable partial dentures (IARPDs) to facilitate retention and support of the prosthesis. Implants are proposed in cases of posterior edentulous areas to convert Kennedy Class I or II to Class III or when the preparation of existing teeth to serve as abutments is contraindicated. The purpose of this report is to describe the protocol applied to fabricate an IARPD to restore a Kennedy Class II mandible of an elderly patient by incorporating traditional methods, such as the altered cast technique. Each step of the clinical procedure is thoroughly illustrated to document the selected appointment sequence. The patient was satisfied with the delivered prosthesis that demanded no additional implants to be placed but exploitation of an existing one. IARPDs are a viable and cost-effective solution substantiated by numerous reports with positive effects on patient satisfaction.

## 1. Introduction

Implant therapy has gone through major advancements since the Branemark era. Nowadays, implants have achieved a survival rate of 96.4%, which can be characterized as satisfactory [1, 2] and has emerged by understanding several biomechanical factors and their effect upon parameters, such as marginal bone loss, which is determined by both local and systemic factors, including age, history of periodontitis, oral hygiene, smoking, implant surface, and prosthesis type [3, 4]. These results have led to the expansion of indications for their use. Implants are currently used not only for the rehabilitation of complete but also for partial edentulism [5].

At first, implants were used for the support of fixed partial dentures (FPDs). The McGill consensus in 2002 was a milestone that established the use of implants for the support of removable prostheses providing improved retention, support, and stability [6]. However, a decrease in the frequency of complete edentulism is expected in the foreseeable future. This fact along with some adverse biomechanical effects of removable partial dentures (RPDs), the rates of inadequate retention and retreatment of this type of prosthesis have led to the incorporation of implants as abutments for RPDs (7).

Implant-assisted RPDs (IARPDs) have been proposed as simple means for lessening the adverse effects of conventional RPDs. The advantages are more evident in cases of posterior edentulism where implant placement can convert a Kennedy Class I or II to Class III [8]. Reduction of base displacement and forces applied upon the abutment teeth are among the advantages of IARPDs [9, 10]. Incorporating implants as abutments negates the need for preparing tooth abutments, thereby facilitating the preservation of healthy dental tissues.

This treatment concept has also shown some biomechanical advantages. According to the literature, tension on terminal abutment teeth is reduced, whereas the pressure on soft tissues is relieved [11]. Denture displacement reduction and possibly less bone resorption and fewer rebasing procedures are also among the benefits of IARPDs [12].

Conventional techniques may also be used for the reduction of denture base displacement. In cases of Kennedy Class I or II, where free-end saddles are present denture bases tend to move towards the mucosa during function. Recording mucosa in its functional form as described in the altered cast technique may improve denture biomechanical behavior in terms of stability and support [13].

The present report aims to describe the protocol applied to fabricate an IARPD to rehabilitate a Kennedy Class II mandible of an elderly patient by incorporating specialized methods, such as the altered cast technique.

## 2. Case Report

**Figure 1:**
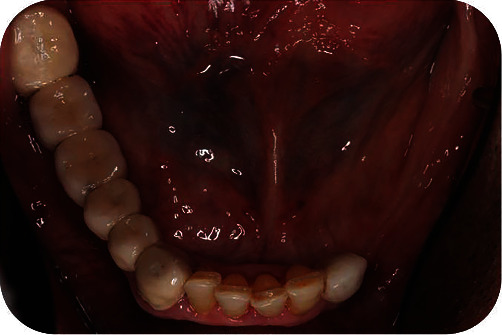
Initial situation. Unilateral posterior edentulousness characterizes the case as a Kennedy Class II.

A 72-year-old male patient presented to the Postgraduate Prosthodontic Clinic of the National and Kapodistrian University of Athens seeking dental rehabilitation. Medical records revealed hypertension, high uric acid levels, and depression, for which the patient received medication. Clinical and radiographic examinations revealed severe loss of dental tissues due to non-carious lesions mainly at the maxilla. The patient was asked to fulfill a 7-day diet diary, which disclosed frequent consumption of acidic food and drinks. The mandible was partially edentulous and characterized as Kennedy Class II. A 6-unit FPD was present at the lower right mandible with teeth #43 and #48 that served as abutments. Poor marginal fit was detected. A dental implant was present in the position of tooth No. #33, whereas the other two implants previously placed posterior to this one had been extracted due to severe periimplantitis (Figure 1).

The patient asked for prosthetic rehabilitation without the placement of additional implants due to his unpleasant previous experience. After careful clinical, radiographic, and laboratory examination of the mounted diagnostic casts, the proposed treatment plan, included fixed prostheses in the maxilla to replace the missing tissues and an IARPD that would utilize the remaining implant, which was characterized with good prognosis, in the mandible. Teeth #43 and #48 would also be used as abutments for the IARPD.

**Figure 2:**
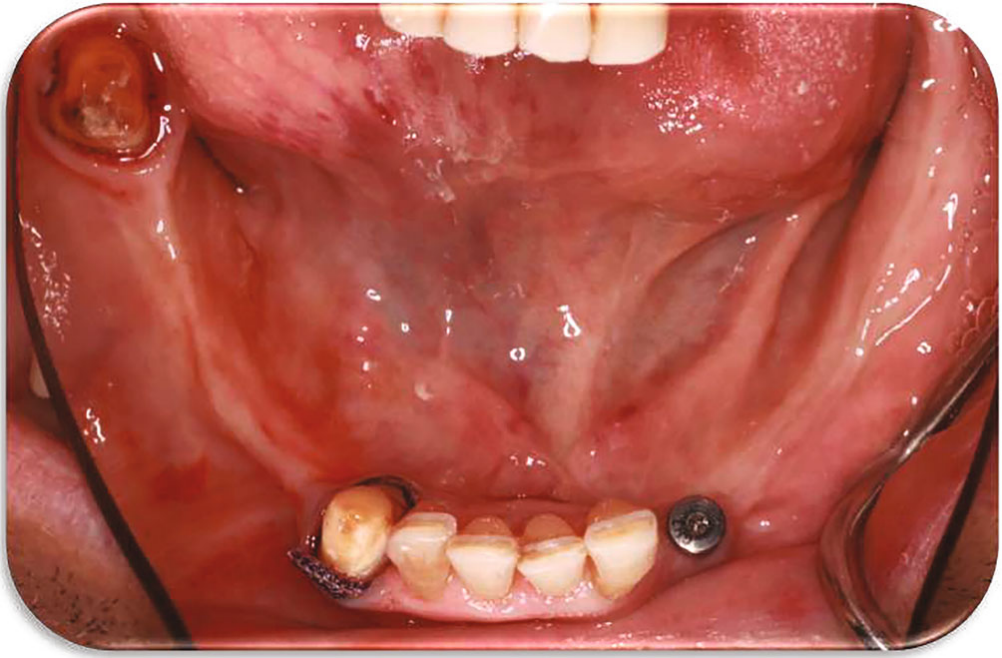
Teeth #43 and #48 were prepared according to the previously defined vertical dimension. A healing abutment was placed on the implant in position No. #33 after the removal of the cement-retained crown.

**Figure 3:**
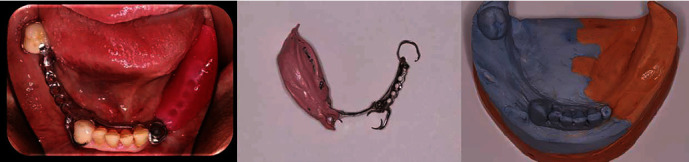
The metal framework carrying a unilateral custom tray at the edentulous area was tried intraorally. Impression of the edentulous area distal to the implant No. #33 was made with the framework in place so that an altered cast can be fabricated.

**Figure 4:**
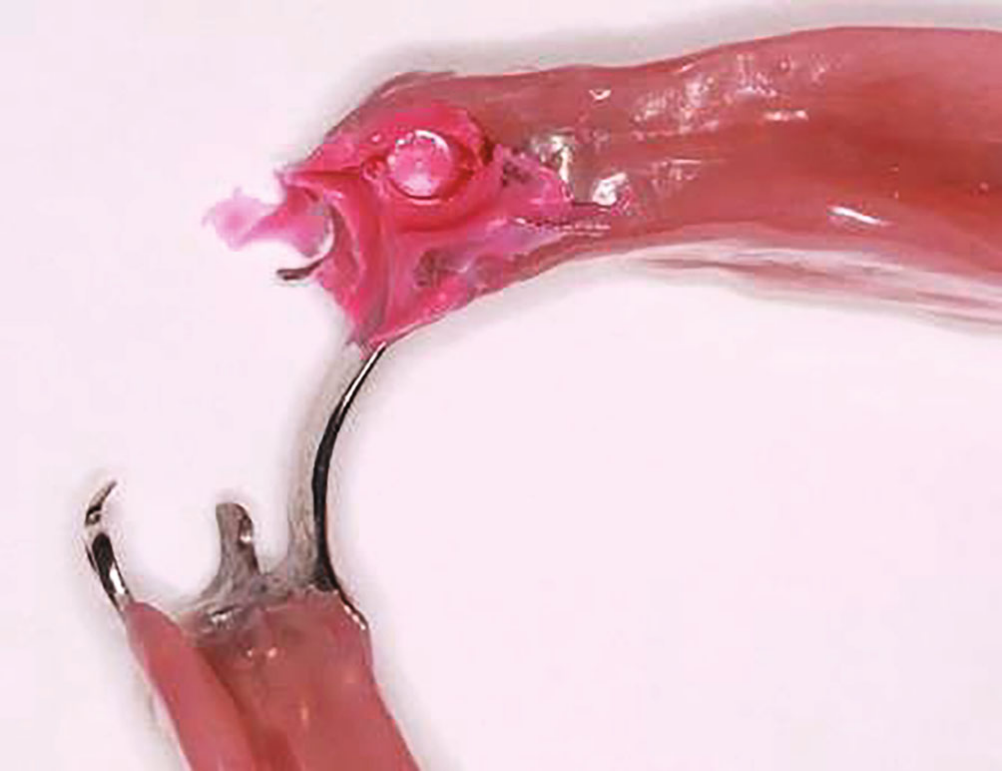
Light flow silicone placement to ensure adequate space presence for the attachment and activation acrylic to be placed.

**Figure 5:**
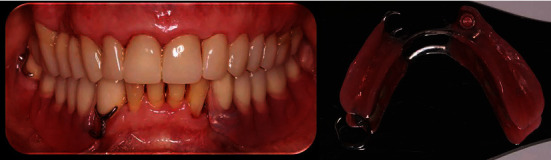
The patient is in the maximum intercuspation position to ensure proper activation of the implant attachment. The activation insert was replaced by one with medium retentive force.

The steps followed for the fabrication of the IARPD were as follows:

1. Existing implant-supported cemented crown on implant No. #33 was removed, and a healing abutment was placed to facilitate periimplant therapy.
2. Initial impressions with an irreversible hydrocolloid material (Hydrogum 5, Zhermack SpA, Germany) were made for a base plate and wax rim to be fabricated for the mandible.
3. Jaw registration at centric relation was performed to define the vertical dimension of occlusion and guide the preparation of teeth #43 and #48 which would serve as abutments for the IARPD (Figure 2).
4. Definite impression of the preparations was made with addition silicone (Zhermack, Zhermack GmbH, Italy) after the application of the immediate dentin sealing technique on the prepared teeth [14].
5. Metal frameworks were checked for their adaptation and marginal fit.
6. The subsequent metal-ceramic crowns were cemented after occlusion check at the bisque try-in appointment.
7. The healing abutment on implant No. #33 was removed and replaced by the connector (locator) after measurement of the gingival height. A stud attachment was selected due to easier handling, and fewer maintenance appointments needed [15].
8. Medium and low viscosity addition silicone (Zhermack, Zhermack GmbH) were used to make an impression with a custom tray. The dental laboratory was asked to modify the metal framework above the implant No. #33 for the subsequent activation of the connector.
9. IARPD's metal framework was ordered to carry a unilateral custom tray at the edentulous area as well as a rest seat on tooth #32 for the correct seating to be checked intraorally during the impression procedure and attachment activation. This extension would be removed at the delivery appointment. An impression of the area distal to the implant No. #33 was made with zinc oxide eugenol cement (SS White Impression Paste, SS White Group, C/O Prima Dental Group, Gloucester, England) after border molding with impression compound (Compound impression Stick, Kerr, KerrHawe S.A., Switzerland). The impression material was chosen due to low dimensional changes, good surface detail reproduction, and adherence to the dental compound. The previous cast was modified according to the altered cast technique [16, 17] (Figure 3).
10. Jaw registrations were performed. The color, size, and morphology of the artificial teeth that would be placed were selected.
11. Teeth set-up try-in was performed to verify previous procedures.
12. Implant attachment activation was performed at the delivery appointment. Because of the instructions previously given to the laboratory, space was created in the acrylic above the stable part of the attachment. This area was filled with low-viscosity silicone, and the IARPD was seated to verify that adequate space was present for the activation (Figure 4).
13. The connected component of the attachment carrying the destined for activation nylon was placed on the stable component. The low-viscosity acrylic resin was placed within the notches of the connected part, and a thicker amount was placed inside the socket on the intaglio surface of the IARPD. After careful insertion of the prosthesis to its correct position, the patient was asked to close his mouth until maximum intercuspation (Figure 5).
14. After complete polymerization of the acrylic, the prosthesis was removed. Any material excess was eliminated and the area was polished. The nylon compartment was replaced by one with the desired retentive force. The IARPD was delivered, and the instructions regarding its proper use and maintenance were given.
15. A recall appointment was scheduled one week later. The patient did not report any discomfort or difficulty using the prosthesis. Frequent recall appointments were set.

## 3. Discussion

**Figure 6:**
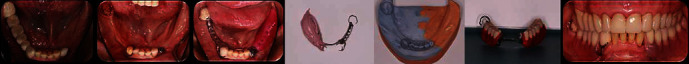
Photo panel depicting clinical and laboratory stages of the IARPD fabrication process utilizing the altered cast technique.

This report presents the step-by-step protocol for the fabrication of an IARPD for a Kennedy Class II mandible of an elderly patient by incorporating specialized methods, such as the altered cast technique (Figure 6). This type of prosthesis exhibits favorable clinical outcomes. According to several studies, oral health-related quality of life (QoL), masticatory ability, and overall satisfaction significantly improve with IARPDs when compared with RPDs, regardless of the chosen attachment type [18]. Moreover, significantly less displacement has been reported for IARPDs compared with conventional dentures [19], an effect, which may be further enhanced by the use of implants with increased diameter and length [20].

Implant placement for the transformation of RPDs to IARPDs entails certain biomechanical advantages. Stresses of chromium–cobalt frameworks in IARPDs are reduced, and the effect is most evident when the implants are placed at the molar instead of the premolar area [21]. The stress on the periodontal membranes of abutment teeth and supporting bone around implants and mucosa decrease with implants placed at first molars sites (22). According to a Finite Element Analysis, implants are expected to bear most of the loading, especially in cases where they are placed in premolar rather than molar areas [23]. However, survival rates of implants used in IARPDs are not inferior to those reported in the literature with a range between 91% and 100% [24]. Marginal bone loss ranges from 0.3 to 2.3 m [24]. The studies report prosthesis survival rates of 90–100% [25].

Technical complications are rare. Plastic components need to be changed every 12 months [26]. However, reduced frequency of rebasing procedures has been reported due to less bone resorption, which is attributed to relieved pressure applied upon soft tissues, especially in cases of thick soft tissue (>2 mm) [11, 12]. The use of different attachment systems does not seem to significantly influence implant survival rate and patient satisfaction [24]. Surveyed crowns resemble conventional designs and constitute a reliable solution [27] but they are demanding in terms of technical difficulty and costs. Stud attachments and especially the ball type are more frequently used as they are considered simple and economical options [24]. Regarding patient satisfaction, they have proven to have a positive effect [28]. One study found that an increase in the implant abutment height leads to the reduction of denture displacement [29].

Studies have underlined that the transformation of a conventional removable prosthesis into an implant-supported one can help patients with benefits in chewing ability, aesthetics, satisfaction, and QoL [30]. Implants in cases of IARPDs are usually placed to convert Kennedy Class I or II to Class III [8]. The presence of distally extended edentulous ridges poses difficulties regarding the fabrication of removable prostheses due to differences in terms of displacement between teeth and mucosa [31]. In cases of RPDs, tooth morphology should be accurately recorded as well as mucosa in its functional form [31]. Even though not widely used due to technical difficulties, expenses, and added time, the altered cast impression technique ensures optimal support and extension. One of the innovations of the described protocol is the incorporation of the altered cast technique [13, 32] within the IARPD fabrication process. Thus, added benefits of the use of implants can be further enhanced with a technique that fulfills the principles of controlled tissue support [31].

The present study comprises a case report, which entails specific limitations. In this case report, an existing implant at the position No. #33 was used to fabricate a Kennedy Class II IARPD by incorporating the altered cast technique. Literature has mainly focused on posteriorly placed implants due to the advantages regarding the reduction of denture displacement. However, there are cases, such as the one described where anterior implants are utilized either because they were previously placed there or because they can only be placed adjacent to the terminal tooth due to severe atrophy of the posterior ridge [33]. Thus, more studies on the stress distribution of IARPDs with anteriorly placed implants are needed.

The altered cast technique is destined for cases of free-end saddles and might not offer additional advantages in cases of true Kennedy Class III or cases where implant placement has transformed a Kennedy Class I or II to Class III. Moreover, the application of the altered cast technique demands experience, skills, and an additional clinical appointment and laboratory stage, which elongates the treatment time and costs.

## 4. Conclusion

The present report aims to describe the protocol applied to fabricate an IARPD to rehabilitate a Kennedy Class II mandible of an elderly patient by incorporating specialized methods, such as the altered cast technique. IARPDs are a justified prosthetic solution for the rehabilitation of partial edentulism. Conversion of Kennedy Class I or II to Class III, avoidance of dental tissue removal through tooth preparations, and adverse effects of conventional RPDs are among the advantages of the treatment of choice and should be considered in cases of medical, dental, or financial constraints. Care should be given so that implants are placed in positions that might later be used in implant-supported fixed prostheses. Benefits introduced with implant placement can be further enhanced with a technique that fulfills the principles of controlled tissue support, such as the altered cast impression technique. Furthermore, research is needed to justify the most suitable implant precision attachment choice in relation to conventional clasps as used in this case report.

## Data Availability

Data supporting this research article are available from the corresponding author or the first author on reasonable request.
